# Microglial reprogramming: a potential new frontier in enhancing immunotherapy for melanoma brain metastasis

**DOI:** 10.1002/1878-0261.70028

**Published:** 2025-03-20

**Authors:** Noam Savion‐Gaiger, Dorin Bar‐Ziv, Harriet Kluger

**Affiliations:** ^1^ Yale Cancer Center Yale School of Medicine New Haven CT USA

**Keywords:** immune checkpoint inhibitors, melanoma brain metastases, microglia, myeloid cells, RelA/NF‐κB, tumor microenvironment

## Abstract

The brain is a common site of metastatic dissemination in advanced melanoma. Due to limited access to samples from human brain metastases, our understanding of the tumor microenvironment in the brain lags behind that of other sites, and murine studies are therefore highly informative. Rodriguez‐Baena et al. conducted elegant studies of myeloid cells within melanoma brain metastases, demonstrating their plasticity and changes before and after colonization by melanoma cells. The immune‐inhibitory changes in microglial cells may be reversed or mitigated by inhibition of RelA/NF‐κB.

## Graphical abstract

Microglia act as tumor suppressors during brain metastasis colonization but shift to a tumor‐promoting role after melanoma brain metastases form. NF‐κB/RelA signaling emerges as a key driver of this phenotypic shift. Targeting this pathway reprograms microglia into a pro‐inflammatory state, enhancing antitumor immunity and immune checkpoint inhibitor responses, offering a novel therapeutic avenue for melanoma brain metastasis.

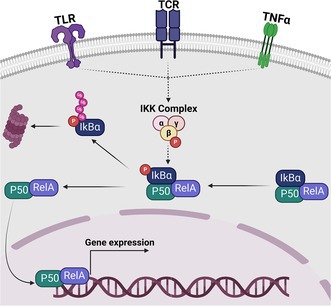

**Abbreviations**
DAM: disease‐associated microglia
DHMEQ: dehydroxymethylepoxyquinomicin
ICI: immune checkpoint inhibitor
MBM: melanoma brain metastases
NF‐κB: nuclear factor kappa B
PIM: pro‐inflammatory microglia
RelA: v‐Rel avian reticuloendotheliosis viral oncogene homolog A
sc‐RNA‐seq: single‐cell RNA sequencing
TAM: tumor‐associated macrophages
TA‐MG: tumor‐associated microglia
TME: tumor microenvironment

## 1 Background and perspective

The spread of melanoma to the brain is challenging if unresponsive to immune checkpoint inhibitors (ICIs) [1]. While innumerable studies have evaluated the tumor microenvironment (TME) in extracranial metastases, it is relatively understudied in the brain due to limited access to human tissue. Small studies demonstrated differences in T‐cell content and tumor vasculature in brain versus extracranial sites in patients treated with ICIs [2, 3]. Single‐cell RNA‐sequencing (sc‐RNA‐seq) technologies have enhanced our understanding, showing that melanoma brain metastases (MBM) are enriched with myeloid cells [4]. Differences between brain‐resident microglia and peripheral myeloid cells have been well‐documented [5]. In MBM, the role of microglia has been examined, with studies demonstrating both protumorigenic and antitumorigenic roles, as reviewed [6].

In a novel study by Rodriguez‐Baena et al. [7], distinct, phenotypically diverse microglial subpopulations were identified. They showed that microglia can transition between pro‐inflammatory/antitumor states and anti‐inflammatory, tumor‐promoting phenotypes in the presence of MBM, largely driven by the RelA/NF‐κB pathway.

## 2 Microglia: Janus‐faced regulators of brain metastasis

Much can be learned about the role of microglia from glioblastomas, where extracranial factors are less important and tumor tissue is often available. Comparisons between primary brain tumors and metastases have shown that divergent myeloid populations have variable effects on T cells and tumor cells, suggesting that myeloid modulation might be preferred to myeloid ablation for optimizing the antitumor immune response [8].

Using two syngeneic murine melanoma models of colonization of the brain, elegant myeloid manipulation methods, and extensive scRNA‐seq of murine MBM, Rodriguez‐Baena identified distinct populations with two major types of tumor‐associated macrophages (TAMs): microglia (expressing Tmem119, P2ry12, Gpr34, Sall1) and nonmicroglial myeloid cells (expressing Lyz2, Itga4, Cd74, Plac8) [7]. Microglia were a more abundant subset within the macrophage population, consistent with studies of the MBM microenvironment [9]. Depleting microglia prior to tumor colonization increased MBM burden but did not alter macrophage or peripheral immune cell infiltration. However, when TAMs were depleted after MBM formation, tumor burden was decreased, indicating that TAMs actively contribute to tumor growth once MBMs are established.

**Fig. 1:**
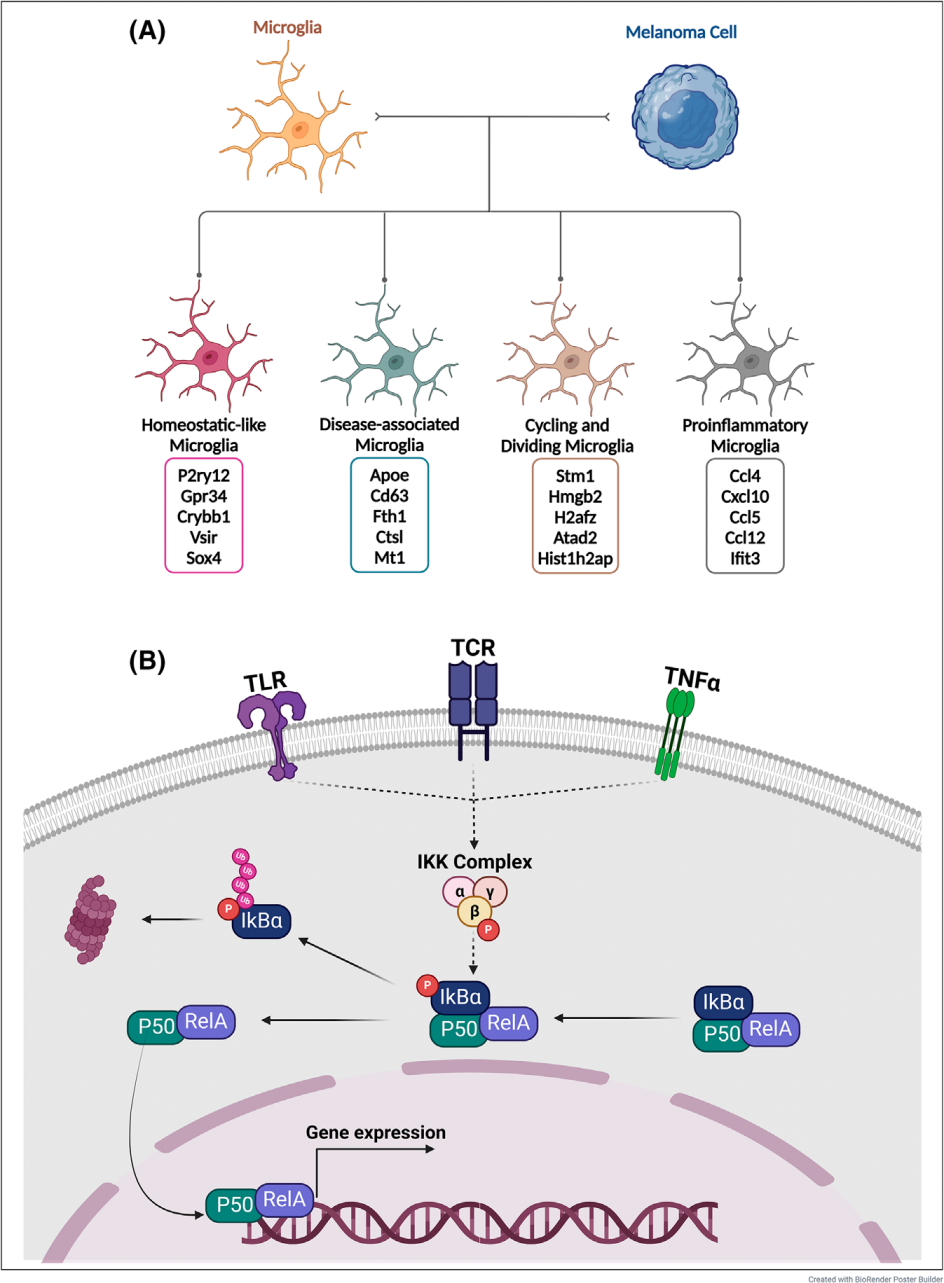
(A) In the presence of melanoma brain metastases, tumor‐associated microglia differentiate into four distinct subpopulations: Homeostatic‐like microglia, characterized by high transcription of genes and pathways associated with glial cell function and homeostasis; Disease‐associated microglia, exhibiting increased translational activity; Proliferating microglia, which include cycling and dividing microglia; and Pro‐inflammatory microglia, marked by enhanced chemokine expression and antigen presentation and strong predicted CD8^+^ and NK cell interactions. RelA deletion in conditional knockout mice resulted in microglia that displayed an enrichment of homeostatic‐like signature and pro‐inflammatory microglial signatures, along with a reduction in disease associated microglia‐like gene transcription. (B) The canonical NF‐κB pathway is activated by pro‐inflammatory cytokines (e.g., TNF‐α, IL‐1), pattern recognition receptors (e.g., TLRs), and antigen receptors. Upon stimulation, the receptor‐associated complex recruits and activates the IκB kinase (IKK) complex, consisting of IKKα, IKKβ, and IKKγ (NEMO). IKKβ phosphorylates IκBα, leading to its ubiquitination and subsequent degradation by the proteasome. This degradation releases the NF‐κB heterodimer, RelA (p65)/p50, allowing it to translocate into the nucleus. Once inside the nucleus, NF‐κB binds to κB‐responsive elements in target genes, promoting the transcription of pro‐inflammatory cytokines, chemokines, and cell survival genes.

Four microglial subpopulations were identified: pro‐inflammatory microglia with enhanced expression of T/NK cell attractant chemokines and antigen presentation genes, disease‐associated microglia with high translational activity, and two clusters linked to glial homeostasis and cell division (Fig. 1A). These important findings still require validation in models of metastasis rather than colonization, as it would be impossible to validate them in humans.

## 3 Microglial NF‐κB activation can enhance brain metastasis growth after colonization

A striking difference between tumor‐associated microglia and nonmicroglial macrophages in the models studied by Rodriguez‐Baena et al. was in their expression of RelA target genes. RelA, a subunit of NF‐κB, is a transcription factor that plays a central role in regulating inflammation, cell survival, and apoptosis signaling in immune cells (Fig. 1B). With RelA depletion after MBM formation, tumor burden was significantly decreased without altering the number of microglia or nonmicroglial macrophages. Administration of dehydroxymethylepoxyquinomicin (DHMEQ), a brain‐penetrant NF‐κB inhibitor, decreased MBM burden and prolonged survival in mice, with no effect on subcutaneous tumors. DHMEQ has challenging pharmacological properties and has not been administered to humans, although it has activity in numerous preclinical models of cancer [10]. However, numerous drugs approved for other purposes inhibit NF‐κB and can potentially be repurposed for treating MBMs [11].

## 4 Targeting NF‐κB reprograms microglia and enhances antitumor immunity

RelA deletion in conditional knockout mice reduced protumorigenic disease‐associated microglia and increased the proportion of pro‐inflammatory microglia, combined with increased T and NK cell‐attracting factors and an influx of CD8^+^ T and NK cells. Given the relatively poor T‐cell infiltration in human MBM and the potential associated lack of response to ICIs, microglial manipulation might provide an avenue to overcome this challenge [12].

NF‐κB modulation for enhancing ICI activity was also studied by Rodriguez‐Baena. While RelA wild‐type mice exhibited a reduced metastatic burden in the *NRas* mutant following anti‐PD‐L1, combining DHMEQ with anti‐PD‐L1 enhanced the intracranial tumor response. Similar findings were demonstrated when combining anti‐PD‐1 with anti‐CTLA‐4. While improvements were seen, effects on survival were not reported, and it is unclear whether any mice had complete tumor rejection, highlighting the differences between murine models and humans, where complete tumor rejection can be seen with ICIs.

## 5 Unanswered questions and future directions

This study provides a deep characterization of myeloid subsets in MBM with solid evidence that NF‐κB pathway activation in microglia drives the transition to a protumor state when MBM are present, suggesting a novel avenue to treat MBM. Previous research on protumorigenic and pro‐inflammatory microglial phenotypes has primarily focused on glioblastoma and brain metastasis from lung and breast cancer. While this study did not compare melanoma to other tumors, further exploration of this phenomenon in other malignancies is warranted, as microglial modulation might depend on interactions with transformed melanocytes, cells of neural crest origin.

The leap between preclinical and clinical studies is often limited by the relevance of the murine models. B16/F10 does not mimic human melanoma genetically, while the *NRas* model reflects the biology of up to 20% of melanomas [13]. Carotid artery injection and intracranial injections guarantee the formation of brain deposits but do not reflect the metastatic process, and these provocative results support the need for further work in models of metastasis, which are challenging, as reviewed [14].

Research on MBM has been hampered by limited human tissue to validate results. Some groups have generated human tissue resources in recent years, providing an opportunity to verify that NF‐κB expression varies in microglia from tumors of responders and nonresponders to ICIs [1].

While microglial subtypes resemble the traditional M1/M2 classification of peripheral macrophages—where M1 are pro‐inflammatory and M2 are pro‐tumorigenic—this binary model fails to fully capture their plasticity. The identification of additional microglial subtypes in this study underscores the complexity of their functional states, necessitating further investigation into their roles within the TME.

These findings have important clinical implications. The evidence provided by Rodriguez‐Baena of the role of NF‐κB in microglia in MBM warrants considering human investigation using novel NF‐κB or existing drugs that might be repurposed [15].

## Conflict of interest

Dr. Kluger has received research grants from Pfizer, Apexigen, and Merck and personal fees from Iovance, Merck, Chemocentryx, Bristol‐Myers Squibb, Signatero, Gigagen, GI reviewers, Pliant Therapeutics, Esai, Invox, Wherewolf, Teva, and Replimmune, all unrelated to this work.

## Author contributions

All three authors contributed to conceptualizing the commentary, and to writing and proofreading the manuscript.
